# The core regulatory network of the abscisic acid pathway in banana: genome-wide identification and expression analyses during development, ripening, and abiotic stress

**DOI:** 10.1186/s12870-017-1093-4

**Published:** 2017-08-29

**Authors:** Wei Hu, Yan Yan, Haitao Shi, Juhua Liu, Hongxia Miao, Weiwei Tie, Zehong Ding, XuPo Ding, Chunlai Wu, Yang Liu, Jiashui Wang, Biyu Xu, Zhiqiang Jin

**Affiliations:** 1 Key Laboratory of Biology and Genetic Resources of Tropical Crops, Institute of Tropical Bioscience and Biotechnology, Chinese Academy of Tropical Agricultural Sciences, Haikou, Hainan China; 2 Key Laboratory of Genetic Improvement of Bananas, Hainan province, Haikou Experimental Station, China Academy of Tropical Agricultural Sciences, Haikou, Hainan China; 3 Hainan Key Laboratory for Sustainable Utilization of Tropical Bioresources, College of Agriculture, Hainan University, Haikou, China

**Keywords:** Abscisic acid signaling, Abiotic stress, Banana, Fruit development and ripening, Gene expression

## Abstract

**Background:**

Abscisic acid (ABA) signaling plays a crucial role in developmental and environmental adaptation processes of plants. However, the PYL-PP2C-SnRK2 families that function as the core components of ABA signaling are not well understood in banana.

**Results:**

In the present study, 24 *PYL*, 87 *PP2C*, and 11 *SnRK2* genes were identified from banana, which was further supported by evolutionary relationships, conserved motif and gene structure analyses. The comprehensive transcriptomic analyses showed that banana *PYL-PP2C-SnRK2* genes are involved in tissue development, fruit development and ripening, and response to abiotic stress in two cultivated varieties. Moreover, comparative expression analyses of *PYL-PP2C-SnRK2* genes between BaXi Jiao (BX) and Fen Jiao (FJ) revealed that *PYL-PP2C-SnRK2*-mediated ABA signaling might positively regulate banana fruit ripening and tolerance to cold, salt, and osmotic stresses. Finally, interaction networks and co-expression assays demonstrated that the core components of ABA signaling were more active in FJ than in BX in response to abiotic stress, further supporting the crucial role of the genes in tolerance to abiotic stress in banana.

**Conclusions:**

This study provides new insights into the complicated transcriptional control of *PYL-PP2C-SnRK2* genes, improves the understanding of *PYL-PP2C-SnRK2*-mediated ABA signaling in the regulation of fruit development, ripening, and response to abiotic stress, and identifies some candidate genes for genetic improvement of banana.

**Electronic supplementary material:**

The online version of this article (doi:10.1186/s12870-017-1093-4) contains supplementary material, which is available to authorized users.

## Background

In plants, phytohormone abscisic acid (ABA) regulates numerous developmental processes, such as seedling development, seed dormancy, and fruit ripening [1–5]. In addition, ABA plays a central role in the adaptation of plants to environmental stresses, such as drought, salinity, and cold [6, 7]. Due to the biological and agricultural importance of ABA, many studies have focused on plant responses to ABA at the level of cytology and molecular biology. Since 2009, the ABA signaling pathway began to be better understood [6]. PYR/PYL/RCARs (ABA receptors), Group A PP2Cs (negative regulators), and SnRK2s (positive regulators) were confirmed as crucial components of ABA signaling in Arabidopsis. Finally, a double negative regulatory model is constituted by these components. SnRK2s activities are repressed by direct dephosphorylation by Group A PP2Cs in the absence of ABA. When responding to developmental or environmental clues, the ABA signal induces PYR/PYL/RCAR interaction with Group A PP2Cs, including ABI1, ABI2, AHG3, and HAB1, leading to inhibition of Group A PP2Cs and activation of SnRK2s [6, 8–10]. This results in phosphorylation or activation of downstream targets, such as ABF/AREB/ABI5, SLAC1, and other ABA-responsive gene products [6, 11]. The ABA-mediated interaction model between PYLs and PP2Cs was validated by in vitro reconstitution in Arabidopsis protoplasts [12].

Additionally, the function of PYL-PP2C-SnRK2 genes in developmental processes and in response to ABA and abiotic stress were characterized in plants. *PYL9*, *PYL5* or *PYL8* overexpression improved drought tolerance or ABA responses in Arabidopsis [9, 13, 14]. In contrast, an ABA insensitive phenotype was observed in the quadruple mutant of pyr1 pyl1 pyl2 pyl4 [10]. Double and triple mutation of several crucial members of Group A *PP2Cs* (*ABI1*, *ABI2*, *HAB1*, *HAB2*, *AHG1*, and *PP2CA*) resulted in enhanced ABA sensitivity, indicating the negative roles of Group A *PP2Cs* in ABA signaling [15–19]. Interference of *AtPP2CA* increased tolerance to freezing stress and ABA sensitivity in Arabidopsis [20]. Mutation of abi2-1 resulted in enhanced tolerance to salt stress and ABA insensitivity in Arabidopsis [21]. Overexpression of *SnRK2.8* improved tolerance to drought stress in Arabidopsis [22]. Conversely, mutation of snrk2.2, snrk2.3, and snrk2.6 decreased drought stress tolerance and ABA responses, such as seed germination, plant growth, stomatal behavior [6]. Besides, the similar roles of *PYL* and *SnRK2* genes were also observed in rice. Overexpression of *OsPYL3* or *OsPYL9* positively regulated the ABA response during seed germination and improved drought and cold stress tolerances in rice [23]. *OsPYL/RCAR5* overexpressing rice plants showed hypersensitivity to ABA during seed germination [24]. Overexpression of *SAPK4* in rice resulted in improved germination, growth and development under salt stress both in seedlings and mature plants [25]. *OsSAPK9* was reported to improve drought tolerance and grain yield through regulating cellular osmotic potential, stomatal closure and stress-responsive gene expression in rice [26]. Interestingly, Arabidopsis plants overexpressing *OsPP108* (a Group A *PP2C* gene in rice) showed highly insensitivity to ABA and tolerance to salt and osmotic stresses during seed germination, root growth and overall seedling growth. This indicated that OsPP108 negatively regulates ABA signaling and positively regulates abiotic stress tolerance [27]. Together, this evidence suggests that Group A *PP2Cs* negatively regulate ABA signaling and negatively/positively regulate ABA-mediated biological processes; and *PYLs* and *SnRK2s* could positively regulate the response of plants to these processes.

To date, genes that encode the crucial components of ABA signaling have been identified in several species based on genome sequencing. There are 14 *PYLs* in Arabidopsis, 13 in rice, 10 in *Selaginella moellendorffi*, and 4 in *Physcomitrella patens*; 9 Group A *PP2Cs* in Arabidopsis, 10 in rice, 5 in *Selaginella moellendorffi*, and 2 in *Physcomitrella patens*; and 10 *SnRK2s* in Arabidopsis, 11 in rice, 6 in *Selaginella moellendorffi*, and 4 in *Physcomitrella patens* [6]. In spite of the economic and social importance of banana and the critical role of *PYL-PP2C-SnRK2s* in the plant development and stress responses, no information is known about the *PYL-PP2C-SnRK2* gene family in banana. Banana is the largest fruit crop and vital for food security for millions of people around the world [28, 29]. Because it is mainly cultivated as a staple food in many impoverished continents, such as Africa, banana studies have proceeded slowly [30]. Investigation of genes in the signal transduction pathways on the basis of complete genome sequences is of benefit for revealing the cellular biological processes [31]. The banana genome sequencing was finished in 2012 [32], which supplies full genome data for us to perform systematic analyses of *PYL-PP2C-SnRK2* gene families.

In this study, we identified 24 *PYLs*, 87 *PP2Cs*, and 11 *SnRK2s* from the banana genome and investigated their phylogenetic relationships, protein motifs, gene structure, and expression patterns in different tissues, in diverse stages of fruit development and ripening, and under abiotic stress. Further, we studied the interaction networks and co-expression profiles of Group A PP2Cs in response to cold, salt, and osmotic stresses. This systematic study increases the understanding of the core components of ABA signaling associated with developmental processes and abiotic stress responses and builds a solid foundation for genetic improvement of banana.

## Results

### Identification and phylogenetic analyses of banana PYL-PP2C-SnRK2s

To identify all PYL-PP2C-SnRK2 family members in banana, both Hidden Markov Model and BLAST searches were carried out to search the banana genome database with PYL-PP2C-SnRK2 sequences from *Arabidopsis* and rice as queries. After confirming their conserved domain using the PFAM and CDD databases, a total of 24 PYL, 87 PP2C, and 11 SnRK2 proteins were identified from the banana genome. The predicted features of the PYL, PP2C and SnRK2 proteins are summarized in Additional file 1: Table S1.

**Fig. 1:**
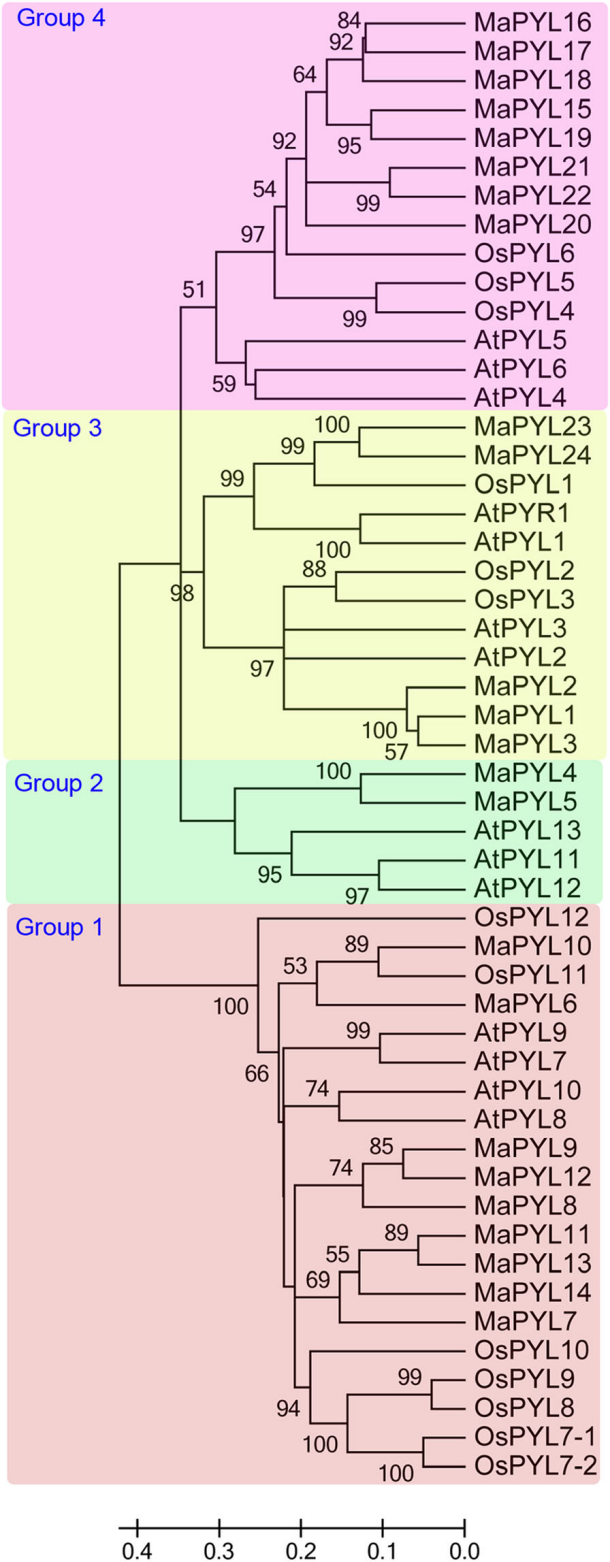
Phylogenetic analysis of PYLs from banana, Arabidopsis, and rice using the complete protein sequences. The Neighbor-joining (NJ) tree was reconstructed using Clustal X 2.0 and MEGA 5.0 softwares with the pair-wise deletion option. 1000 bootstrap replicates were used to assess tree reliability

**Fig. 2:**
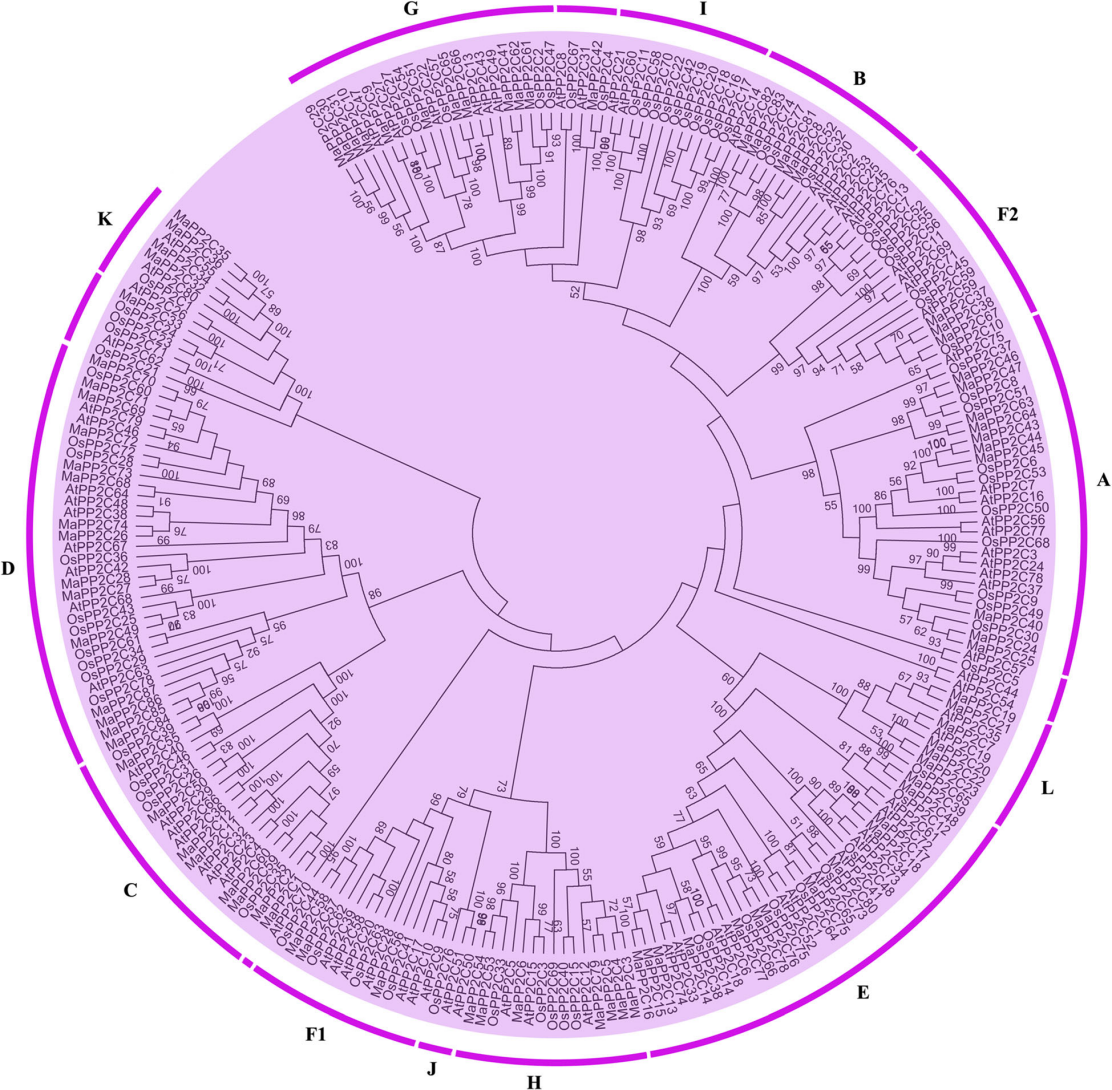
Phylogenetic analysis of PP2Cs from banana, Arabidopsis, and rice using the complete protein sequences. The Neighbor-joining (NJ) tree was reconstructed using Clustal X 2.0 and MEGA 5.0 softwares with the pair-wise deletion option. 1000 bootstrap replicates were used to assess tree reliability

**Fig. 3:**
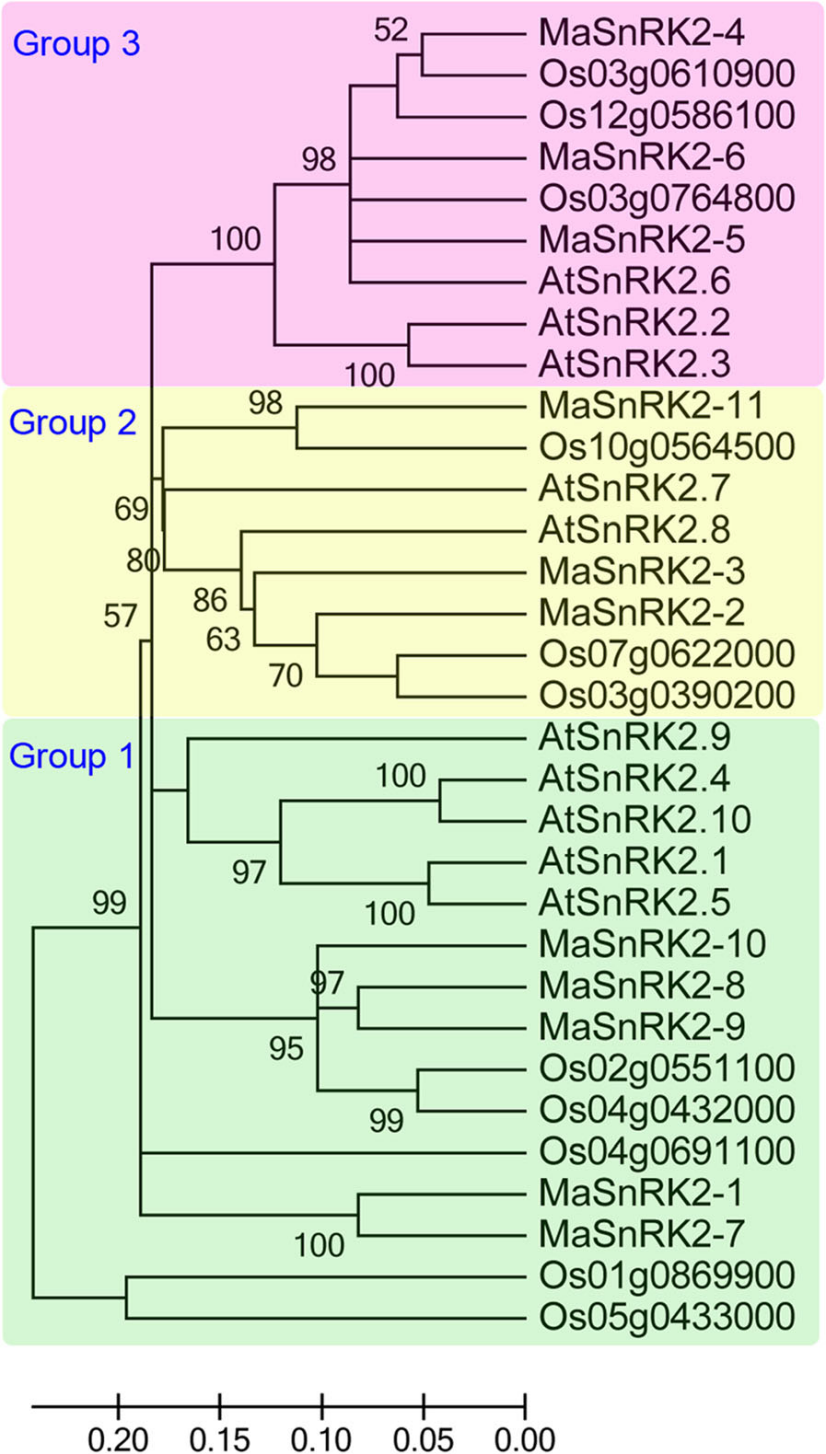
Phylogenetic analysis of SnRK2s from banana, Arabidopsis, and rice using the complete protein sequences. The Neighbor-joining (NJ) tree was reconstructed using Clustal X 2.0 and MEGA 5.0 softwares with the pair-wise deletion option. 1000 bootstrap replicates were used to assess tree reliability

To understand the phylogenetic relationship of PYL-PP2C-SnRK2 proteins, neighbor-joining (NJ) trees were reconstructed with the complete PYL-PP2C-SnRK2 protein sequences from banana, *Arabidopsis* and rice (Figs. 1, 2, and 3). According to the phylogenetic analyses, the PYL, PP2C, and SnRK2 families were divided into 4 (group 1-4), 13 (group A-L), and 3 (group 1-3) subgroups, respectively. Some orthologous PYL-PP2C-SnRK2s between banana and rice were identified, which implied that some ancestral PYL-PP2C-SnRK2s existed prior to the divergence of banana and rice. Generally, banana PYL-PP2C-SnRK2s showed closer relationships with PYL-PP2C-SnRK2s in rice than those in Arabidopsis, which is accordance with the current understanding of plant evolutionary history.

### Conserved motifs and gene structure analyses of banana PYL-PP2C-SnRK2

**Fig. 4:**
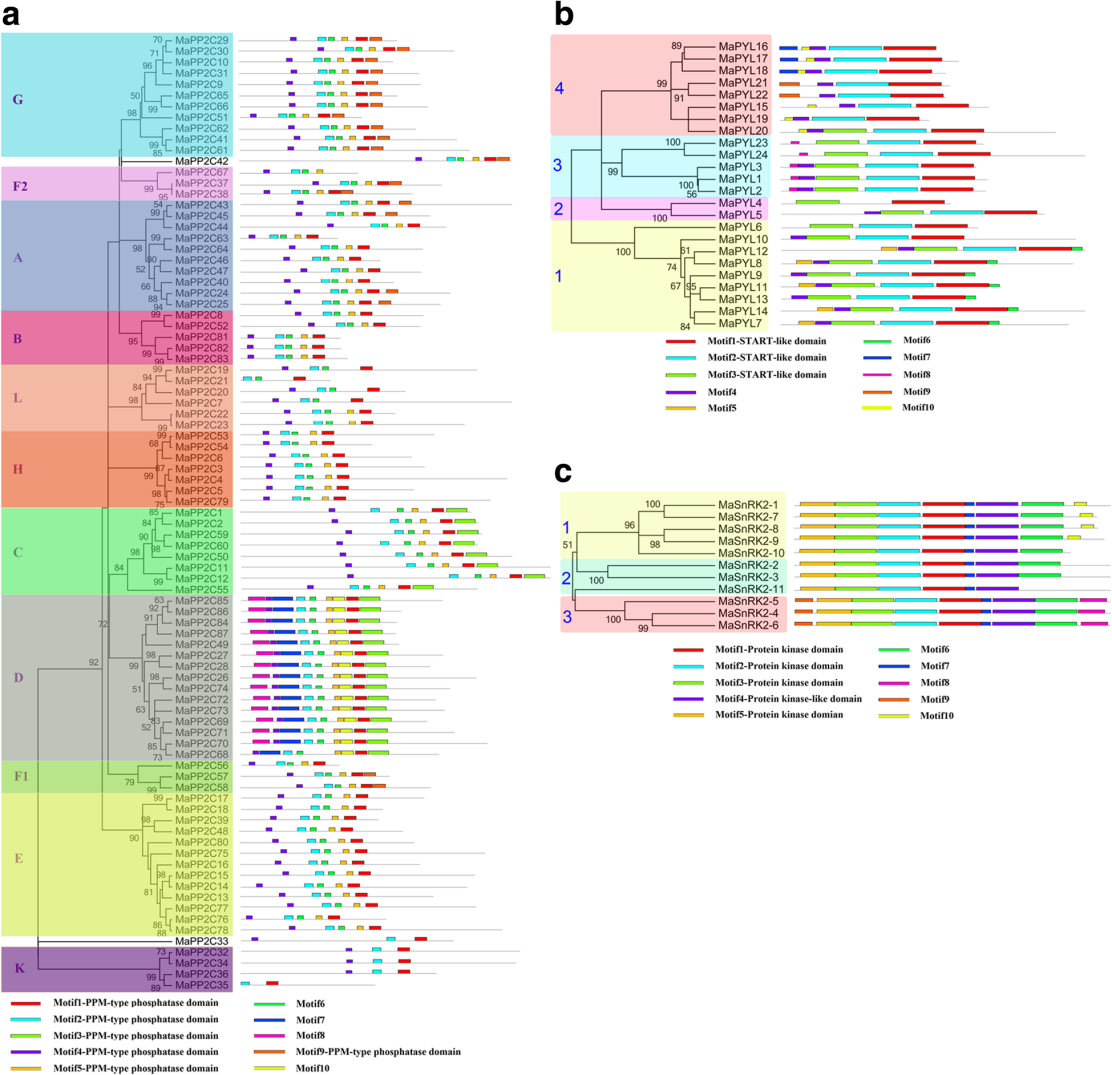
The conserved motifs of banana PP2Cs (**a**), PYLs (**b**), and SnRK2s (**c**) according to phylogenetic relationship. All motifs were identified by MEME database with the complete amino acid sequences of banana PP2Cs, PYLs, and SnRK2s

To get insight into the structural features of the banana PYL-PP2C-SnRK2 proteins, conserved motifs were analyzed based on the phylogenetic relationship. Ten conserved motifs were acquired for each gene family with MEME and InterPro databases (Fig. 4). For the banana PYL family, motifs 1-3 were annotated as the START-like domain. All the identified MaPYLs contained motifs 1 and 2. The subgroup 1-3 also showed the conserved motif 3 (Fig. 4b). For the banana PP2C family, motifs 1-5 were annotated as the PPM-type phosphatase domain. Almost all of the PP2Cs contain the motifs 1, 2, 4, and 5, except for subgroup K showing motifs 1, 2, and 4. Interestingly, subgroup C specially showed motif 3, and subgroup D uniquely had motif 3, 7, 8, and 10 (Fig. 4a). For the banana SnRK2 family, motifs 1-5 were annotated as the Protein kinase domain. All the MaSnRK2s have motifs 1-5. Motif 10 was especially pronounced in subgroup 1 and motifs 8 and 9 were only found in subgroup 3 (Fig. 4c). This indicates that all the identified PYL-PP2C-SnRK2s have typical family features and the proteins classified into the same subgroup share similar amino acid sequences.

**Fig. 5:**
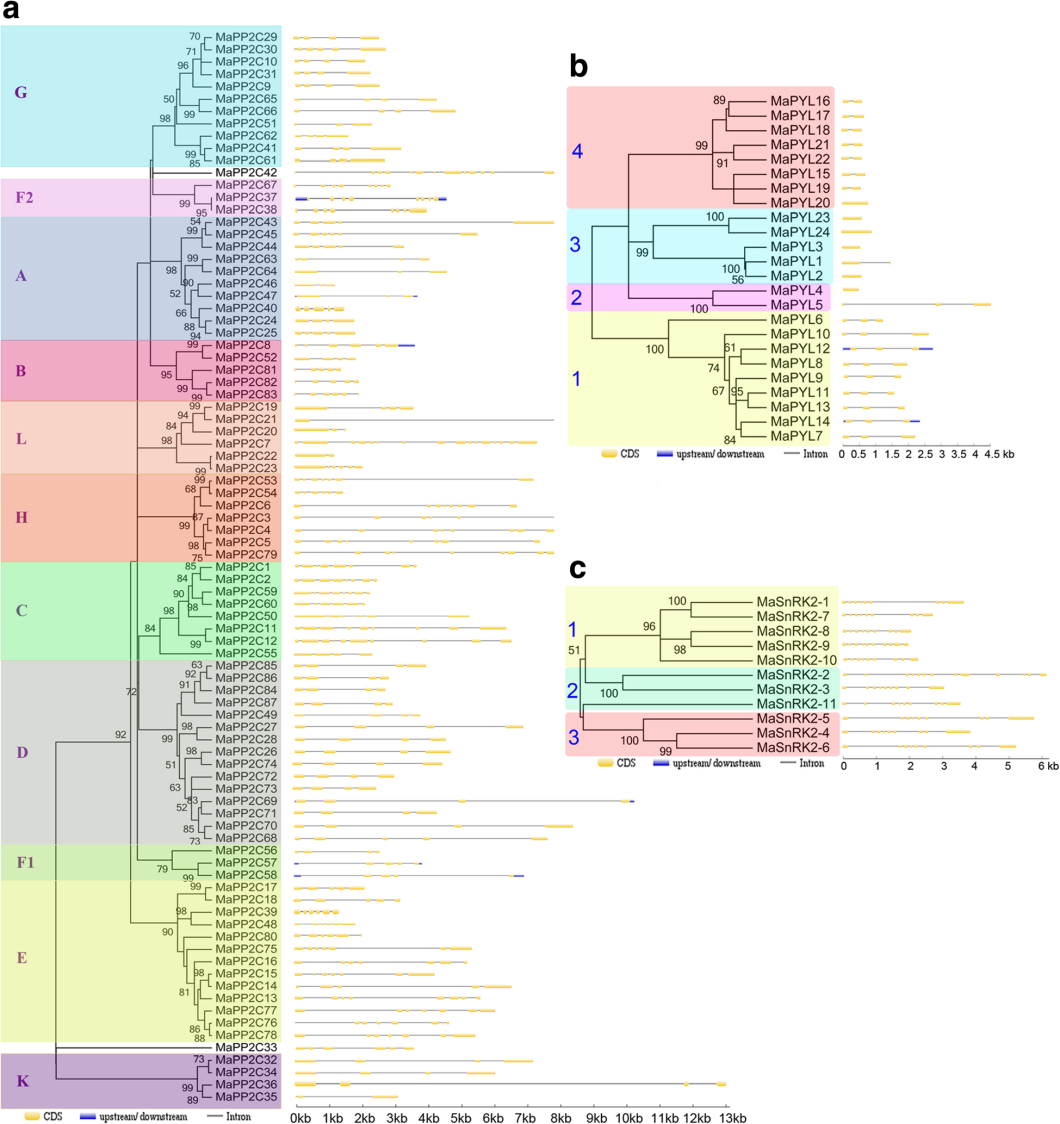
Gene structure analyses of banana *PP2Cs* (**a**), *PYLs* (**b**), and *SnRK2s* (**c**) according to phylogenetic relationship. Exon-intron structure analyses were performed by GSDS database. The *blue boxes*, *yellow boxes*, and the *black lines* indicate upstream/downstream, exons, and introns, respectively

To better understand the gene structure of banana *PYL-PP2C-SnRK2s*, exon-intron organizations of these genes were tested (Fig. 5). For the banana *PYL* family, subgroups 1, 3, and 4 have 2, 0, and 1 introns, respectively; and subgroup 2 showed 0-2 introns (Fig. 5b). For the banana *PP2C* family, subgroups A, B, D, F1, G, and K contain 2-5 introns; subgroups C, E, F2, and H have 3-9 introns; and subgroup L shows 1-15 introns (Fig. 5a). For the banana *SnRK2* family, subgroups 1, 2, and 3 show 8-9, 8-13, and 8 introns, respectively (Fig. 5c). These results indicate that *PYL-PP2C-SnRK2* genes in the same subgroup show similar exon-intron organization.

### Expression analyses of *PYL-PP2C-SnRK2* genes in different banana tissues

**Fig. 6:**
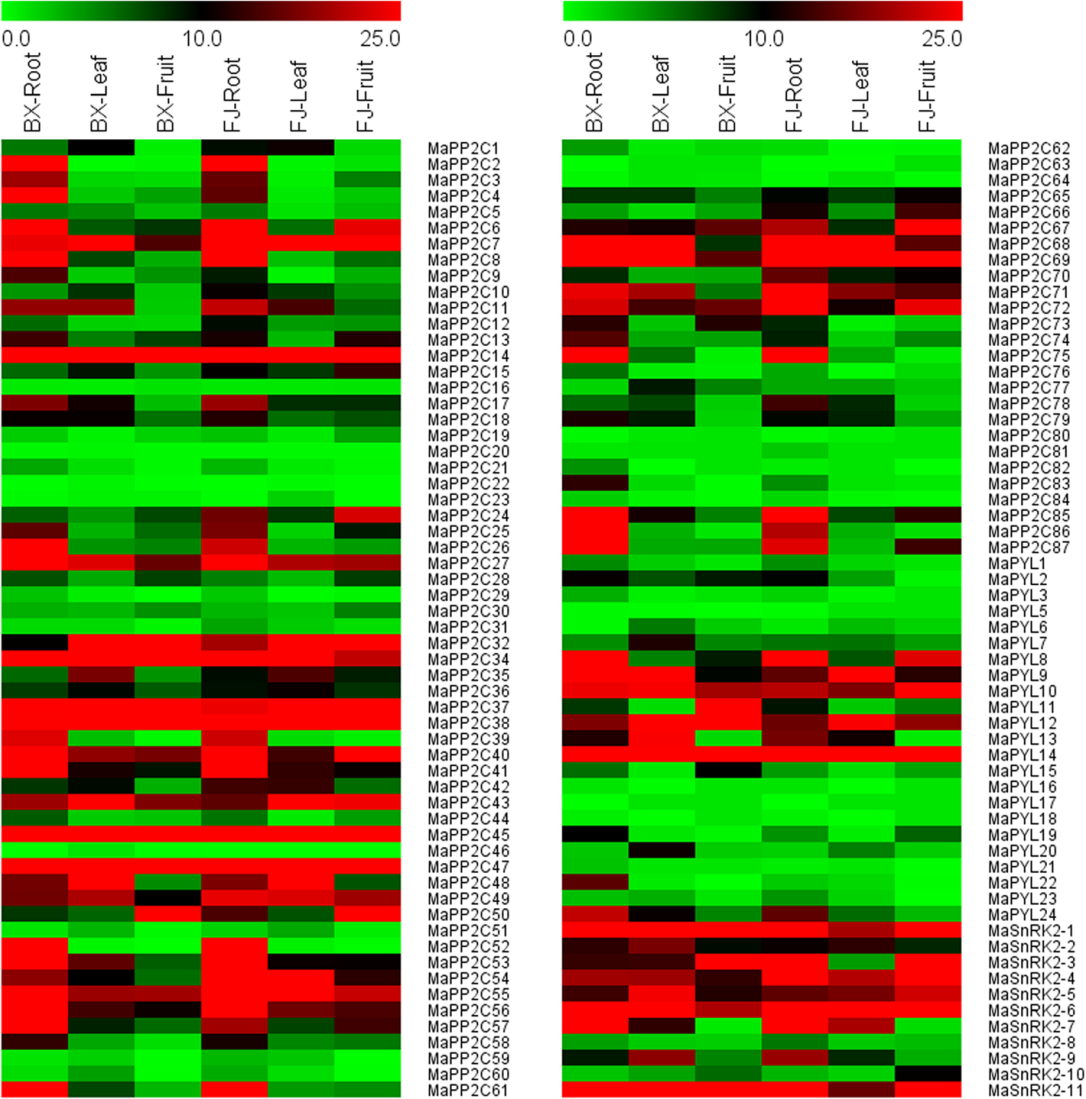
Expression profiles of banana *PP2Cs*, *PYLs*, and *SnRK2s* in roots, leaves, and fruits of BX and FJ. The heat map was constructed according to the FPKM value of banana *PP2Cs*, *PYLs*, and *SnRK2s* from two independent experiments. FPKM value is shown in color as the scale

To examine the expression profiles of *PYL-PP2C-SnRK2* genes in different tissues of banana, roots, leaves, and fruits from BaXi Jiao (*Musa acuminate* L. AAA group cv. Cavendish, BX) and Fen Jiao (*Musa* ABB PisangAwak, FJ) were collected to perform trancriptomic assays (Fig. 6; Additional file 1: Tables S2; S3; S4; S5). Generally, most of the *PYL-PP2C-SnRK2* genes showed similar tissue expression patterns between BX and FJ. For example, several genes (*MaPYL-14, MaPP2C-14, −34, −37, −38, −45, −47, and MaSnRK2-6*) displayed high transcript abundance (FPKM value > 20) in both BX and FJ. In contrast, some genes (*MaPYL-5, −16, −17, −18, −21*, and *MaPP2C-16, −20, −22, −23, −29, −46, −59, −63, −64, −80, −81, −84*) had low transcript abundance (FPKM value < 3) in both BX and FJ.

In addition, we also found different expression patterns of *PYL-PP2C-SnRK2* genes between BX and FJ. For the *PYL* family, the number of genes with high expression levels (FPKM value > 10) in roots and leaves was greater in BX (10/22 and 8/21, respectively) than in FJ (7/22 and 5/20, respectively). For the *PP2C* family, the number of genes with high expression levels (FPKM value > 10) in roots and fruits was less in BX (48/86 and 19/82, respectively) than in FJ (51/86 and 33/84, respectively). This phenomenon was also observed in the tissue expression patterns of the *SnRK2* family. Taken together, the tissue expression patterns of *PYL-PP2C-SnRK2* genes in two cultivated varieties could lay a foundation for further investigation of tissue development and function.

### Expression analyses of *PYL-PP2C-SnRK2* genes in different stages of fruit development and ripening

**Fig. 7:**
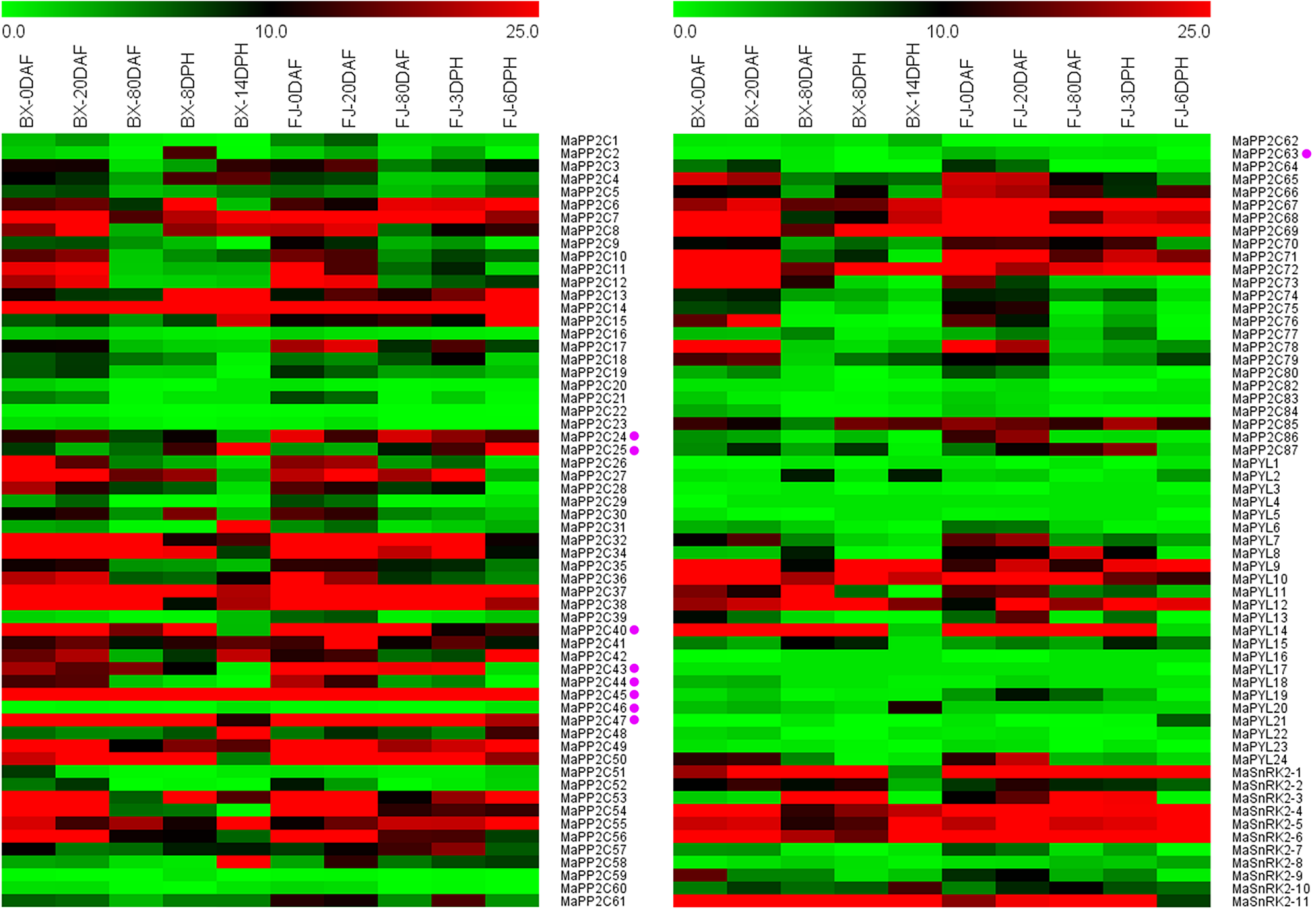
Expression profiles of banana *PP2Cs*, *PYLs*, and *SnRK2s* in different stages of fruit development and ripening in BX and FJ varieties. The heat map was constructed according to the FPKM value of banana *PP2Cs*, *PYLs*, and *SnRK2s* from two independent experiments. FPKM value is shown in color as the scale. Group A *PP2Cs* are marked with purple dot

To get some clues on the function of the *PYL-PP2C-SnRK2* genes in fruit development and ripening of banana, total RNA was extracted during different stages of fruit development and ripening for transcriptomic analyses (Fig. 7; Additional file 1: Tables S6; S7; S8; S9).

According to the transcriptomic data, most of *PYL-PP2C-SnRK2* genes showed similar expression patterns at different stages of fruit development and ripening in both BX and FJ. Some genes showed high expression levels (FPKM value > 10) at different stages of fruit development and ripening. For the *PYL* family, 7/22, 7/22, 5/16, 4/19, and 4/17 *PYL* genes showed high expression levels (FPKM value > 10) at 0 days after flower (DAF), 20 DAF, 80 DAF, 8 days post-harvest (DPH), and 14 DPH in BX, respectively; and 7/21, 9/22, 5/17, 5/17, and 3/19 *PYL* genes showed high expression levels (FPKM value > 10) at the corresponding stages in FJ, respectively. For the PP2C family, 47/85, 45/87, 19/82, 30/83, and 27/79 *PP2C* genes showed high expression levels (FPKM value > 10) at 0 DAF, 20 DAF, 80 DAF, 8 DPH, and 14 DPH in BX, respectively; and 51/85, 52/85, 33/84, 35/84, and 28/82 *PP2C* genes showed high expression levels (FPKM value > 10) at the corresponding stages in FJ, respectively. For the SnRK2 family, 6/11, 6/11, 6/11, 7/11, and 5/10 *SnRK2* genes showed high expression levels (FPKM value > 10) at 0 DAF, 20 DAF, 80 DAF, 8 DPH, and 14 DPH in BX, respectively; and 6/11, 7/11, 7/11, 6/11, and 4/11 SnRK2 genes showed high expression levels (FPKM value > 10) at the corresponding stages in FJ, respectively. These results indicated the possible involvement of *PYL-PP2C-SnRK2* genes in banana development and ripening.

The number of *PP2C* genes in BX with high expression levels (FPKM value > 10) was more at 0 (47/85) and 20 (45/87) DAF than at subsequent stages, including 80 DAF (19/82), 8 DPH (30/83), and 14 DPH (27/79). Also, similar expression patterns for *PP2C* genes were observed in FJ. These results indicate that *PP2C* genes play an important role during early fruit development.

Notably, FJ showed more *PYL* genes with high expression levels (FPKM value > 10) than BX at 20 DAF and 3 DPH. *PP2C* genes with high expression levels (FPKM value > 10) were more in FJ than in BX during all the tested stages, except for 6 DPH. More *SnRK2* genes with high expression levels (FPKM value > 10) was also observed in FJ relative to BX at 20 and 80 DAF. These results imply that *PYL-PP2C-SnRK2* genes may be more active in FJ than in BX during fruit development and ripening stages.

A total of 17 *PYL-PP2C-SnRK2* genes, including *MaPYL-9, −10, −12, MaPP2C-7, −14, −32, −37, −45, −47, −49, −55, −67, −69, −72*, and *SnRK2-4, −5, −6*, showed high expression levels (FPKM value > 10) during all the tested stages in both BX and FJ, indicating the extensive and vital role of these genes during fruit developmental and ripening processes.

Most of the Group A *PP2Cs*, including *PP2C-24, −40, −43, −45,* and *−47*, showed high expression levels (FPKM value > 10) in the majority of the development and ripening stages of BX and FJ, whereas *PP2C-16, −20, −22, −23,* −*46, −59, −60, −62, −63, −82,* and −83 had extremely low expression (FPKM value < 3) during all the stages of fruit developmental and ripening in both BX and FJ. In addition, 8, 4, 7, 7, 8 Group A *PP2C* genes showed higher expression levels (FPKM value > 10) in FJ than in BX at each stages, respectively.

### Expression analyses of *PYL-PP2C-SnRK2* genes in response to cold, salt, and osmotic stresses

**Fig. 8:**
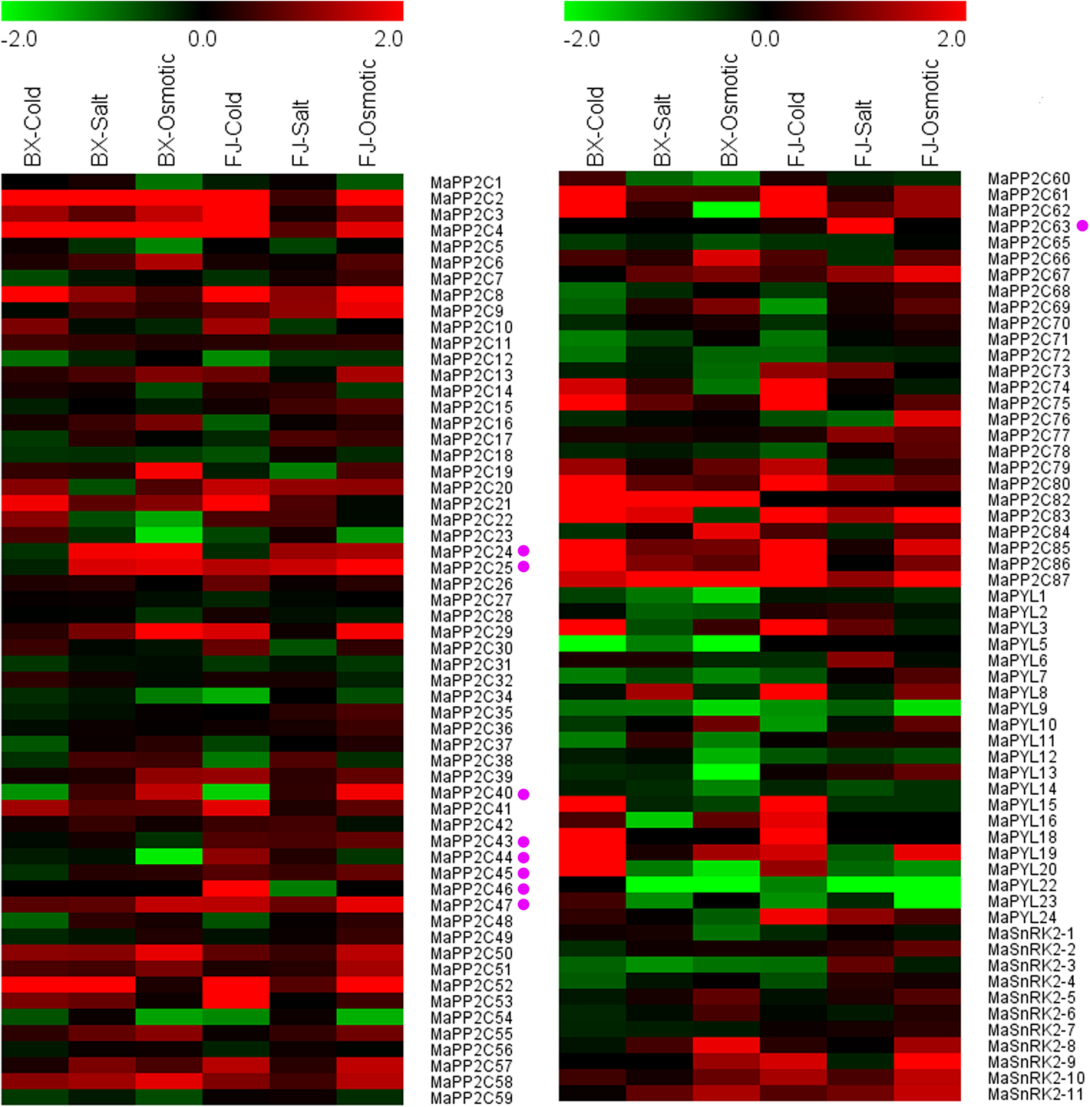
Expression profiles of banana *PP2Cs*, *PYLs*, and *SnRK2s* in response to cold, salt, and osmotic treatments in BX and FJ varieties. Log2 based fold change was used to create the heat map. Fold changes in gene expression are shown in color as the scale. Group A *PP2Cs* are marked with purple dot

To gain insight into the role of *PYL-PP2C-SnRK2* genes in banana in response to abiotic stress, the leaves of banana after cold, salt, and osmotic treatments were collected for transcriptomic analyses (Fig. 8; Additional file 1: Tables S10; S11; S12; S13).

Under the cold treatment, 4/21 *PYLs*, 17/84 *PP2Cs*, and 0/11 *SnRK2s* showed significant upregulation (Log2 based fold change >1; *P*-value < 0.05) in BX, whereas 5/21 *PYLs*, 19/84 *PP2Cs*, and 2/11 *SnRK2s* were significantly upregulated in FJ. Under the salt treatment, 1/21 *PYLs*, 10/84 *PP2Cs*, and 0/11 *SnRK2s* showed significant induction in BX, while 1/21 *PYLs*, 6/84 *PP2Cs*, and 0/11 *SnRK2s* were significantly upregulated in FJ. Under the osmotic treatment, 1/21 *PYLs*, 10/84 *PP2Cs*, and 1/11 *SnRK2s* were significantly induced in BX, whereas 1/21 *PYLs*, 21/84 *PP2Cs* and 2/11 *SnRK2s* were significantly upregulated in FJ. These results suggest that the number of *PYL-PP2C-SnRK2* genes upregulated by cold and osmotic stresses was more in FJ than in BX, implying that these genes may be more active in FJ than in BX in response to cold and osmotic stresses.

Notably, 2 *PYL* genes (*MaPYL8* and *MaPYL15*) and 12 *PP2C* genes (*MaPP2C-3, −4, −21, −52, −53, −61, −62, −74, −75, −85, −86,* and *−87*) were strongly induced (Log2 based fold change >2; *P*-value < 0.05) after cold treatment in FJ. Six *PP2C* genes (*MaPP2C-2, −8, −25, −52, −83,* and *−87*) and 1 SnRK2 genes (*MaSnRK2-11*) were strongly upregulated (Log2 based fold change >2; *P*-value < 0.05) by osmotic treatments in FJ. These genes may be crucial candidates for further use to improve abiotic stress tolerance of banana.

In addition, 10 genes (*MaPYL-8, −24, MaPP2C-20, −39, −47, −52, −53, −57,* and *MaSnRK2-9, −10*), 5 genes (*MaPYL24* and *MaPP2C-67, −77, −80, −83*), and 14 genes (*MaPP2C-87, −83, −67, −52, −57, −61, −85, −25, −9, −8, −51, −13, −76* and *SnRK2-9*) were significantly induced (Log2 based fold change >1; *P*-value < 0.05) by cold, salt, and osmotic treatments, respectively in FJ, but were not significantly induced in BX. These results indicate that these genes may uniquely function on the tolerance of FJ to abiotic stress.

Several Group A *PP2Cs* showed different expression patterns between BX and FJ in response to abiotic stress. *MaPP2C-25, −43, −44, −45, −46,* and *−63* were upregulated in FJ after cold treatment, whereas in BX, were downregulated or did not show any change. *MaPP2C-44* and *−63* showed upregulation in FJ after salt treatment, whereas downregulation or no change in BX. *MaPP2C43* showed induction in FJ after osmotic treatment, but showed repression in BX.

### PYL-PP2C-SnRK2 interaction networks and their co-expression after abiotic stress treatment

**Fig. 9:**
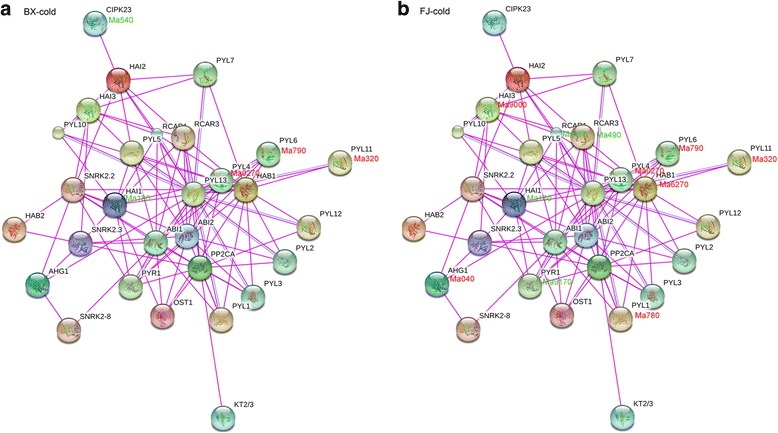
Interaction network and co-expression analyses of Group A *PP2Cs* after cold treatments in BX (**a**) and FJ (**b**) and related genes in Arabidopsis. The genes marked with *red* show upregulation (Log2 based fold change >1). The genes marked with *green* show downregulation (Log2 based fold change < −1)

**Fig. 10:**
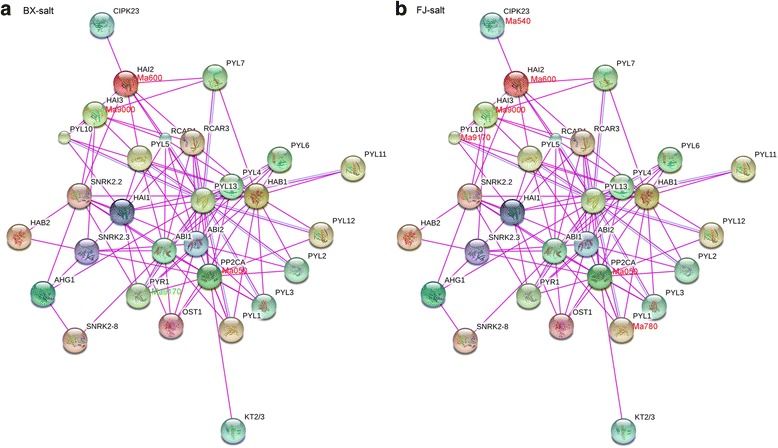
Interaction network and co-expression analyses of Group A *PP2Cs* after salt treatments in BX (**a**) and FJ (**b**) and related genes in Arabidopsis. The genes marked with *red* show upregulation (Log2 based fold change >1). The genes marked with *green* show downregulation (Log2 based fold change < −1)

**Fig. 11:**
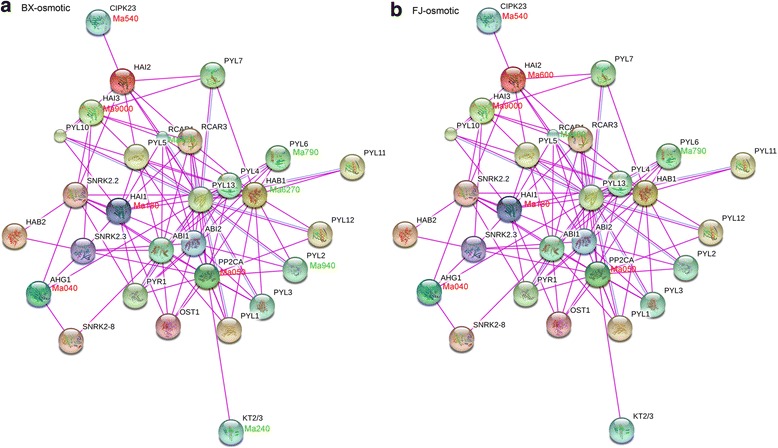
Interaction network and co-expression analyses of Group A *PP2Cs* after osmotic treatments in BX (**a**) and FJ (**b**) and related genes in Arabidopsis. The genes marked with *red* show upregulation (Log2 based fold change >1). The genes marked with *green* show downregulation (Log2 based fold change < −1)

To better understand the biological function of PYL-PP2C-SnRK2s in banana, the possible interaction networks and co-expression of Group A banana PP2Cs were investigated based on experimentally validated interactions of Group A PP2Cs in Arabidopsis and transcriptomic data in banana (Figs. 9, 10 and 11; Additional file 1: Table S14). Firstly, an Arabidopsis Group A PP2C-mediated interaction network was created and 29 interactive proteins (with high confidence; score > 0.9), including 9 PP2Cs and 20 other interactive proteins, were identified with STRING. Secondly, homologs of these interacting proteins in banana were identified with reciprocal BLASTP analyses. Lastly, the expression profiles of the banana genes in BX and FJ under abiotic stress were extracted from RNA-seq data sets.

Under the cold and salt treatments in BX, no gene pair was found to be co-expressed (Figs. 9a and 10a). Under the osmotic treatment in BX, gene pairs HAB1:Ma6270-PYL2:Ma940/PYL6:Ma790/RCAR1:Ma460 showed uniform downregulation (Fig. 11a). Under the cold treatment in FJ, gene pairs HAB1:Ma6270-PYL4:Ma0270/PYL6:Ma790/PYL11:Ma320/PYL1:Ma780 had upregulated co-expression, whereas HAI1:Ma130-PYR1:Ma9170/RCAR1:Ma460/RCAR3:Ma490 showed co-expression of uniform downregulation (Fig. 9b). Under the salt treatment in FJ, gene pairs HAI2:Ma600- CIPK23:Ma540/PYL10:Ma9170, HAI3:Ma9000- PYL10:Ma9170, and PYL1:Ma780-PP2CA:Ma050 showed uniform upregulation (Fig. 10b). Under the osmotic treatment in FJ, HAI2:Ma600- CIPK23:Ma540 had upregulated co-expression (Fig. 11b). Collectively, these results suggest that more gene pairs were uniformly upregulated in FJ than in BX under cold, salt, and osmotic treatments, indicating the crucial roles of Group A PP2C-mediated network in stress signaling.

## Discussion

ABA signaling plays a crucial role in regulating developmental processes and in adaptation to environmental stresses in plants [6, 7]. Investigation of the core regulatory network in the ABA pathway would advance the understanding of the roles of ABA signaling and the function of ABA-associated genes. Currently, no information is known regarding the *PYL-PP2C-SnRK2* gene family in banana. Herein, a total of 24 PYLs, 87 PP2Cs, and 11 SnRK2s were identified from the banana genome, which was classified into 4, 13, and 3 subgroups respectively according to phylogenetic relationship (Figs. 1, 2, and 3). This classification is in accordance with previous phylogenetic analyses of PYL, PP2C, or SnRK2s in Arabidopsis, rice, *Brassica napus*, and *Brachypodium distachyon* [6, 33–35]. Moreover, the phylogenetic classification of PYL-PP2C-SnRK2 was also supported by conserved motif anslysis (Fig. 4). Conserved motif analyses showed that all the PYLs, PP2Cs, and SnRK2s had START-like, PPM-type phosphatase, and protein kinase domains, respectively, and each subfamily shared similar motifs. These typical characteristics of PYL-PP2C-SnRK2s were also observed in other plant species, such as Arabidopsis, apple, and *Brachypodium distachyon* [6, 7, 35, 36].

As one of the most popular fruits, fruit development and ripening process are crucial for banana fruit quality. ABA signaling has been demonstrated to participate in the fruit development process and ripening of many plant species, including sweet cherries, strawberry, and tomato [2–5]; however, whether *PYL-PP2C-SnRK2s* participate in fruit development and post-harvest ripening of banana is unclear. In the present study, we found that more than 4/19 *MaPYLs*, 19/82 *MaPP2Cs*, and 5/10 *MaSnRK2s* showed high expression levels (FPKM value >10) in BX at any one stage of fruit development and ripening. Also, in FJ, more than 3/19 *MaPYLs*, 28/82 *PP2Cs*, and 4/11 *MaSnRK2s* showed high expression levels (FPKM value >10) at any one stage of fruit development and ripening (Fig. 7; Additional file 1: Tables S6; S7; S8; S9). Moreover, a total of 17 *PYL-PP2C-SnRK2* genes, including *MaPYL-9, −10, −12, MaPP2C-7, −14, −32, −37, −45, −47, −49, −55, −67, −69, −72*, and *SnRK2-4, −5, −6*, showed high expression levels (FPKM value > 10) during all the tested stages in both BX and FJ. Considering the negative role of PP2C in ABA signaling, we also found 11 *MaPP2C* genes (*PP2C-16, −20, −22, −23,* −*46, −59, −60, −62, −63, −82,* and −83) showing extremely low expression (FPKM value < 3) during all the stages of fruit developmental and ripening in both BX and FJ. These results imply that *PYL-PP2C-SnRK2* genes may be involved in the fruit development and ripening processes of banana.

The number of *PP2C* genes with high expression levels (FPKM value > 10) was more at 0 and 20 DAF than at subsequent stages in both BX and FJ, implying their regulatory role during early fruit development (Fig. 7; Additional file 1: Tables S6; S7; S8; S9). This is consistent with the expression of *CsPP2C1* that reached the first peak value at early stages during cucumber fruit development [37].

Accumulated evidences suggests that exogenous application of ABA could accelerate fruit ripening of banana [38]; however, the role of the core components of ABA signaling, *PYL-PP2C-SnRK2*, in banana development and ripening is unknown. By comparing the *PYL-PP2C-SnRK2* expression profiles at different stages of fruit development and ripening between BX and FJ, an interesting phenomenon was observed. The number of *PYL-PP2C-SnRK2* genes with high expression levels (FPKM value > 10) was more in FJ than in BX at several stages, which implied that *PYL-PP2C-SnRK2* genes may be more active in FJ than in BX during fruit development and ripening stages (Fig. 7; Additional file 1: Tables S6; S7; S8; S9). Previously, we observed that FJ ripened faster than BX during postharvest ripening. It took 8 and 14 DPH to reach more green than yellow and full yellow degrees of ripening for BX, respectively, whereas it only took 3 and 6 DPH for FJ, respectively [28, 29]. In tomato, RNA interference-mediated repression of ABA biosynthesis resulted in delay of fruit senescence and extension of shelf life [39]. In strawberry, inhibition of *FaNCED1* led to a significant decrease of ABA levels and delay of fruit ripening by gene silencing and RNA interference [40]. In grape, fruit development and quality were improved by exogenous application of ABA [41]. This evidence demonstrates that ABA signaling plays a positive role in fruit development and ripening. Additionally, down-regulation of the *FaPYR1* gene significantly delayed fruit ripening and repressed the expression of ABI1 and SnRK2 genes in strawberry, which implied that *PYL-PP2C-SnRK2* genes may positively regulate fruit development and ripening [42]. Therefore, these findings suggest that *PYL-PP2C-SnRK2*-mediated ABA signaling may contribute to fruit development and ripening in banana.

Because banana has shallow roots, permanent green canopy, and rapid growth rate, it is usually subjected to water stress caused by abiotic stress such as cold, drought, or salt [43]. Investigation of the mechanism underlying banana response to abiotic stress is of great importance for banana breeding. Although ABA plays a predominant role in regulating plants’ tolerance to abiotic stress, the role of the core components of ABA signaling, *PYL-PP2C-SnRK2*, in banana responding to abiotic stress is unknown. In the present study, we found that many *PYL-PP2C-SnRK2* genes showed transcriptional changes after cold, salt, or osmotic treatment in both BX and FJ, indicating that these genes may function on the regulation of banana tolerance to abiotic stress (Fig. 8; Additional file 1: Tables S10; S11; S12; S13).

By comparing the expression patterns of *PYL-PP2C-SnRK2* genes under abiotic stress between BX and FJ, it was clear that more genes were significantly upregulated (Log2 based fold change >1) in FJ than in BX under the cold and osmotic treatments (Fig. 8; Additional file 1: Tables S10; S11; S12; S13). Furthermore, from the interaction network and co-expression analyses, more gene pairs were uniformly upregulated in FJ than in BX in response to the osmotic, cold, and salt stresses (Figs. 9, 10, and 11; Additional file 1: Table S14). The B-genome has been considered to be related to tolerance to abiotic stresses. The banana species *M. balbisiana* with the B-genome is demonstrated to have strong resistance to drought or water stress [44, 45]. Moreover, the “ABB” banana genotypes are more tolerant to drought and other abiotic stresses than other genotypes [46]. Thus, the banana varieties based on the “ABB” genotype can be used as a crucial genetic resource for crop improvement for abiotic stress. FJ (ABB genotype), containing the B-genome, has been reported to have strong tolerance to abiotic stress [28, 29]. Much evidence confirms that *PYL-* and *SnRK2-*mediated ABA signaling play a positive role in plants response to abiotic stress [6, 9, 13, 14, 22]. Together, these findings suggest that more *PYL-PP2C-SnRK2* genes and gene pairs upregulated by abiotic stress in FJ could contribute to the tolerance of banana to abiotic stress.

Previously, Group A *PP2Cs* were demonstrated to be negative factors of ABA signaling [6, 27], whereas the function of Group A *PP2Cs* in ABA-mediated biological processes seem to be different in different species [20, 21, 27]. For example, mutation of *abi2-1* resulted in enhanced tolerance to salt stress in Arabidopsis [21], while Arabidopsis plants overexpressing *OsPP108* showed increased tolerance to salt and osmotic stresses [27]. Most of the Group A *PP2Cs* displayed high expression levels during fruit development and ripening in tomato [18]. Moreover, most of the Group A *PP2C* members were induced at transcriptional levels under osmotic, cold, salt, and drought treatments in Arabidopsis [47]. Based on our transcriptomic data, most of the Group A *PP2Cs* showed high expression levels (FPKM value > 10) in the majority of the development and ripening stages of BX and FJ, and Group A *PP2C* genes were found to be more active in FJ than in BX at transcriptional levels after cold, salt, and osmotic treatments. The function and mechanism of *PP2Cs* in ABA signaling transduction and ABA-mediated biological processes need to be further clarified in future studies.

## Conclusions

In this study, we identified 24 *PYL*, 87 *PP2C*, and 11 *SnRK2* genes from banana and studied their classification and evolutionary relationships by evolutionary, conserved protein motif, and gene structure analyses. The expression analyses reveal the involvement of *PYL-PP2C-SnRK2* genes in banana fruit development, ripening, and responses to abiotic stress. Additionally, comparison of the differential expression profiles of *PYL-PP2C-SnRK2* genes between BX and FJ suggested that *PYL-PP2C-SnRK2*-mediated ABA signaling might positively regulate banana fruit ripening and responses to abiotic stress. Furthermore, interaction networks and co-expression assays demonstrated the strong transcriptional response of core components of ABA signaling in FJ responding to abiotic stress, further supporting the crucial role of the genes for banana tolerance to abiotic stress. These data will supply abundant information for functional characterization of *PYL-PP2C-SnRK2* genes, advance the understanding of *PYL-PP2C-SnRK2*-mediated ABA signaling in the regulation of fruit development, ripening, and response to abiotic stress, and lay a solid foundation for further research on banana breeding.

## Methods

### Plant materials and treatments

Two banana cultivars of BaXi Jiao (*Musa acuminate* L. AAA group cv. Cavendish, BX) and Fen Jiao (*Musa* ABB PisangAwak, FJ) were used in this study. BX is widely planted in China due to its virtues of long storage and high production. FJ is widely cultivated in the Hainan province of China. FJ has stronger tolerance to abiotic stress, including drought, salt, and cold, and ripened faster than BX during postharvest ripening (unpublished data). BX and FJ seedlings at the five-leaf stage were acquired from the banana tissue culture center (Institute of Bananas and Plantains, Chinese Academy of Tropical Agricultural Sciences, Danzhou). Seedlings with consistent growth state were cultured in soil under the conditions of 70% relative humidity and 200 μmol m^−2^ s^−1^ light intensity in 16 h light/8 h dark cycle, 28 °C. Roots and leaves from the five-leaf stage plants, and fruits of 80 DAF were sampled for expression analysis in different organs. Fruits from 0 DAF (budding), 20 DAF (cutting flower) and 80 DAF (harvest stage) were collected to study the expression profiles of genes during fruit development process. Fruits from 8 DPH and 14 DPH in BX and 3 DPH and 6 DPH in FJ were sampled to investigate gene expression patterns during post-harvest ripening stages because FJ reach full yellow degree faster than BX after harvesting [28, 29]. Banana seedlings at the five-leaf stage were irrigated with 200 mM mannitol or 300 mM NaCl for 7 days to study gene expression in response to osmotic and salt stresses, respectively. Banana seedlings were incubated in 4 °C for 22 h to detect gene expression upon cold stress.

### Identification and phylogenetic analyses

The whole protein sequences of banana were downloaded from the banana genome database [32]. The PYL, PP2C, and SnRK2 protein sequences from rice and *Arabidopsis* were obtained from RGAP and UniProt databases, respectively [48, 49]. The HMM profiles built from the known PYL-PP2C-SnRK2s were used as queries to search the banana dataset with HMMER software [50, 51]. BLAST was also employed to identify the predicted banana PYL-PP2C-SnRK2s with all PYL-PP2C-SnRK2s from rice and *Arabidopsis* as queries. Then, the conserved domains of predicted banana PYL-PP2C-SnRK2s were further confirmed with PFAM and CDD databases [52, 53]. The accession numbers of identified banana PYLs, PP2Cs, and SnRK2s are displayed in Table S1. The phylogenetic tree was reconstructed with the PYL-PP2C-SnRK2 proteins from Arabidopsis, rice, and banana using MEGA 5.0 and Clustal X2.0 softwares (bootstrap values for 1000 replicates) [54, 55].

### Protein properties and sequence analyses

Using the ExPASy database, the isoelectric points and molecular weights of the banana PYL-PP2C-SnRK2s were predicted [56]. MEME software was used to identify motifs of banana PYL-PP2C-SnRK2 proteins, and then the motifs were annotated with InterProScan [57, 58]. The optimum width of motifs ranged from 6 to 50, the maximum number of motifs was 10, and the other parameter settings used were default values. The *PYL-PP2C-SnRK2* gene structure was analyzed by GSDS [59]. With the help of STRING software, the Group A PP2Cs-mediated protein interactions in Arabidopsis were explored with the confidence score > 0.9 and no more than 20 interactors.

### Transcriptomic analysis

Total RNA of each sample was extracted with plant RNA extraction kit (TIANGEN, China) and used for cDNA library construction. The sequencing was performed with an Illumina GAII following manufacturer’s instructions. Using FASTX-toolkit, adapter sequences in the raw sequence reads were removed. After examining the sequence quality and removing low quality sequences by FastQC, clean reads were generated. Using Tophat v.2.0.10, clean reads were maped to the DH-Pahang genome (*Musa acuminate*, A-genome, 2n = 22) [32]. The transcriptome assemblies were performed by Cufflinks [60]. The RNA-seq reads status was listed in Additional file 1: Tables S2; S6; S10. Genes were scored as not expressed if the corresponding RNA-seq reads could not align to the genome. Calculation the ratio of *PYL-PP2C-SnRK2* genes with high expression levels or showing significantly changes after abiotic stress treatments was performed according to the genes that is expressed. Gene expression levels were calculated as Reads Per Kilobase of exon model per Million mapped reads (FPKM). DEGseq was used to identify differentially expressed genes (Log2 based fold change >1 or Log2 based fold change <−1; *P*-value < 0.05) in response to cold, salt, and osmotic stresses [61]. There are two biological replicates, which showed good consistency (Additional file 2: Figures S1; S2; S3; Additional file 1: Table S3; S7; S11).

## Additional files

Additional file 1: Table S1. Characteristics of banana PYL, PP2C, and SnRK2 gene families. **Table S2.** Properties of transcriptome for RNA-seq analysis in different tissues. **Table S3.** FPKM value from each biological replicates of PYL-PP2C-SnRK2 genes in different tissues. **Table S4.** Expression data of the banana PYL-PP2C-SnRK2 genes in different tissues of BX and FJ varieties. **Table S5.** Statistical analyses of the expression data related to banana PYL-PP2C-SnRK2 genes in different tissues of BX and FJ varieties. **Table S6.** Properties of transcriptome for RNA-seq analysis in different stages of fruit development and ripening. **Table S7.** FPKM value from each biological replicates of PYL-PP2C-SnRK2 genes in different stages of fruit development and ripening. **Table S8.** Expression data of the banana PYL-PP2C-SnRK2 genes in different stages of fruit development and ripening in BX and FJ varieties. **Table S9.** Statistical analyses of the expression data related to banana PYL-PP2C-SnRK2 genes in different stages of fruit development and ripenings of BX and FJ varieties. **Table S10.** Properties of transcriptome for RNA-seq analysis under cold, salt, and osmotic treatments. **Table S11.** FPKM value from each biological replicates of PYL-PP2C-SnRK2 genes in response to cold, salt, and osmotic stresses. **Table S12.** Expression data (log2-based value) of the banana PYL-PP2C-SnRK2 genes after various abiotic stress treatment in BX and FJ. **Table S13.** Statistical analyses of the expression data related to banana PYL-PP2C-SnRK2 genes in response to abiotic stress. **Table S14.** Expression data of the genes involved in Group A PP2C-mediate interaction networks under abiotic stress in BX and FJ varieties. **Table S15.** The accession numbers and gene name of PYL-PP2C-SnRK2 gene families in Arabidopsis and rice. (XLS 216 kb)

Additional file 2: Figure S1. Expression profiles of banana *PP2Cs*, *PYLs*, and *SnRK2s* in roots, leaves, and fruits of BX and FJ. The heat map was constructed according to the FPKM value of banana *PP2Cs*, *PYLs*, and *SnRK2s* from each replicates of two independent experiments. **Figure S2.** Expression profiles of banana *PP2Cs*, *PYLs*, and *SnRK2s* in different stages of fruit development and ripening in BX and FJ varieties. The heat map was constructed according to the FPKM value of banana *PP2Cs*, *PYLs*, and *SnRK2s* from each replicates of two independent experiments. **Figure S3.** Expression profiles of banana *PP2Cs*, *PYLs*, and *SnRK2s* in response to cold, salt, and osmotic treatments in BX and FJ varieties. The heat map was constructed according to the FPKM value of banana *PP2Cs*, *PYLs*, and *SnRK2s* from each replicates of two independent experiments. (PDF 1249 kb)

## Abbreviations

ABA: Abscisic acid
BX: BaXi Jiao
DAF: Days after flower
DPH: Days post-harvest
FJ: Fen Jiao
FPKM: Reads Per Kilobase of exon model per Million mapped reads
NJ: Neighbor-joining
