# Supplementary material for: The core regulatory network of the abscisic acid pathway in banana: genome-wide identification and expression analyses during development, ripening, and abiotic stress
Source: BMC Plant Biol. 2017 Aug 29;17:145. doi: 10.1186/s12870-017-1093-4 (PMC5576091; doi:10.1186/s12870-017-1093-4)
Supplement: Supplementary file 2 — Expression profiles of banana PP2Cs, PYLs, and SnRK2s in roots, leaves, and fruits of BX and FJ. The heat map was constructed according to the FPKM value of banana PP2Cs, PYLs, and SnRK2s from each replicates of two independent experiments. Figure S2. Expression profiles of banana PP2Cs, PYLs, and SnRK2s in different stages of fruit development and ripening in BX and FJ varieties. The heat map was constructed according to the FPKM value of banana PP2Cs, PYLs, and SnRK2s from each replicates of two independent experiments. Figure S3. Expression profiles of banana PP2Cs, PYLs, and SnRK2s in response to cold, salt, and osmotic treatments in BX and FJ varieties. The heat map was constructed according to the FPKM value of banana PP2Cs, PYLs, and SnRK2s from each replicates of two independent experiments. (PDF 1249 kb) [file 12870_2017_1093_MOESM2_ESM.pdf]

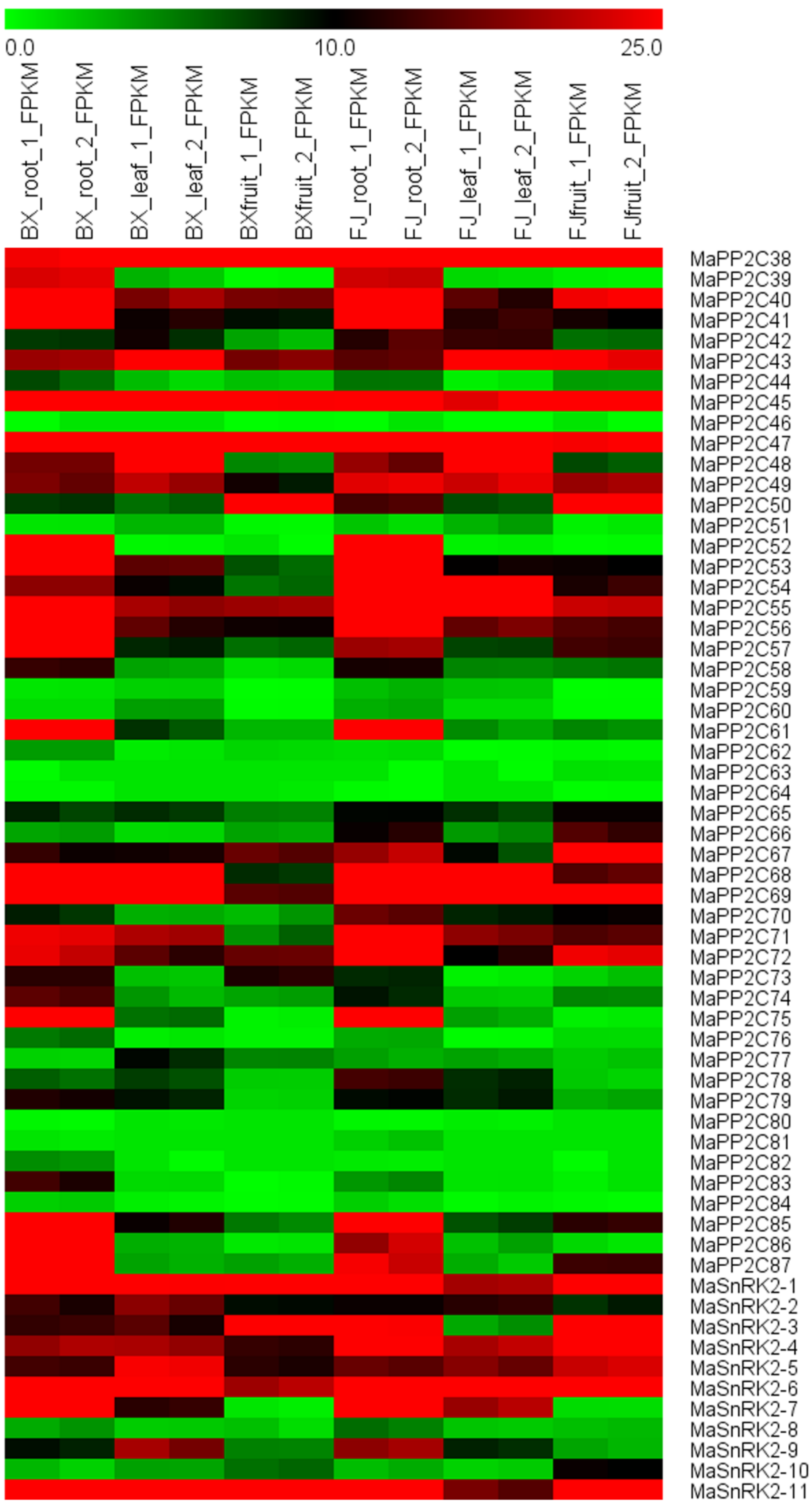

Additional file 2: Figure S1. Expression profiles of banana *PP2Cs*, *PYLs*, and *SnRK2s* in roots, leaves, and fruits of BX and FJ. The heat map was constructed according to the FPKM value of banana *PP2Cs*, *PYLs*, and *SnRK2s* from each replicates of two independent experiments.

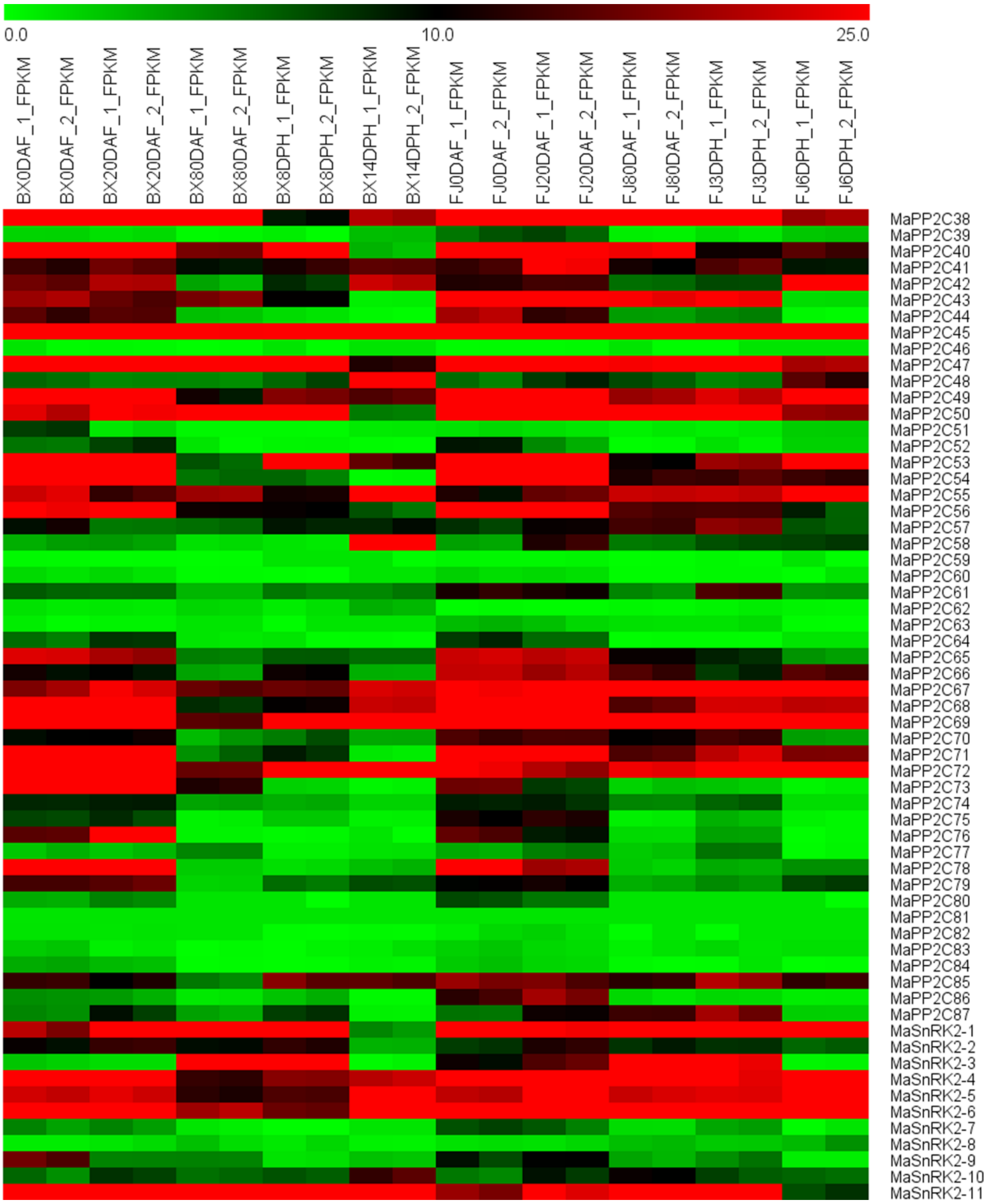

Additional file 2: Figure S2. Expression profiles of banana *PP2Cs* , *PYLs*, and *SnRK2s* in different stages of fruit development and ripening in BX and FJ varieties. The heat map was constructed according to the FPKM value of banana *PP2Cs*, *PYLs*, and *SnRK2s* from each replicates of two independent experiments.

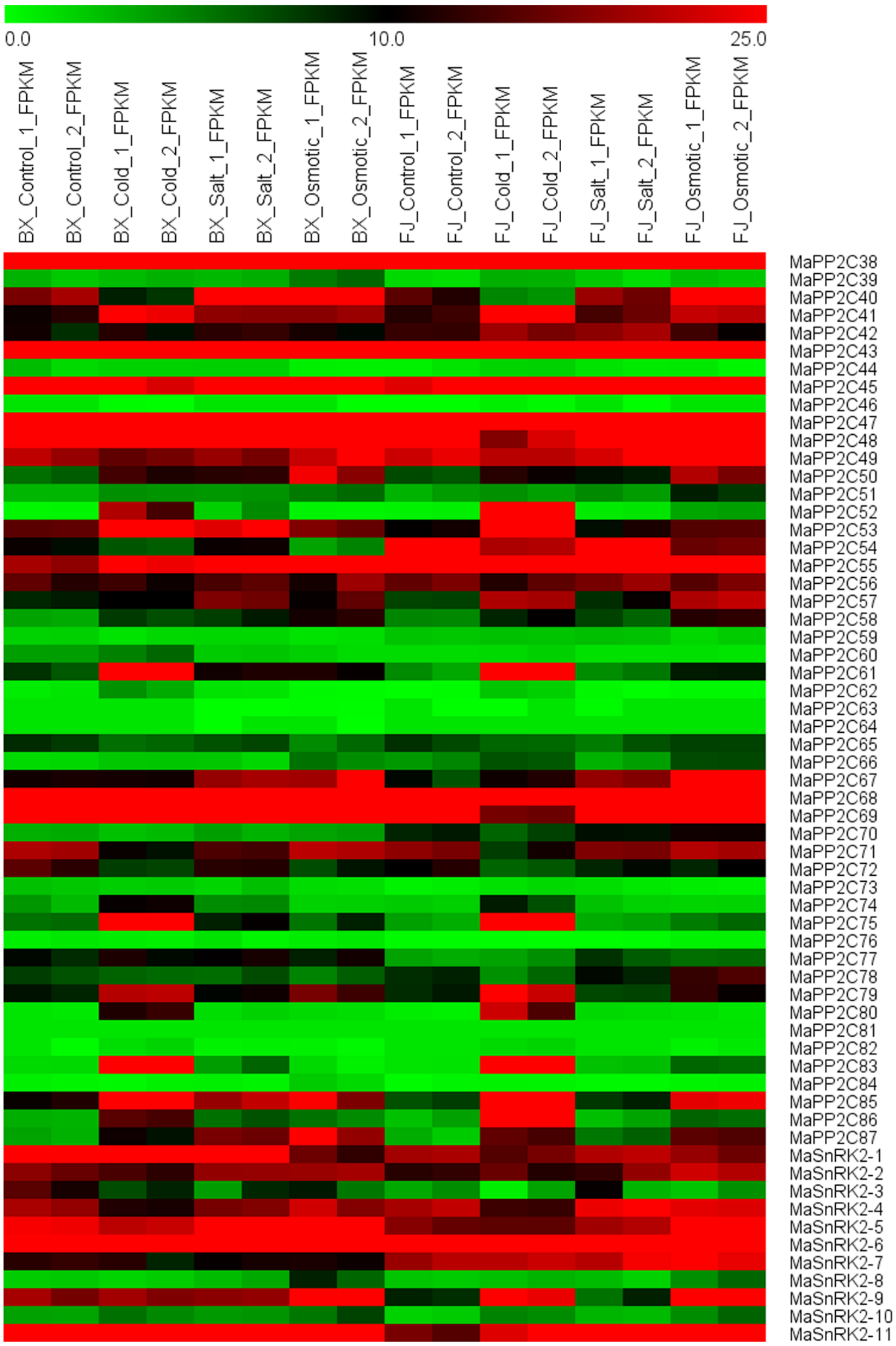

Additional file 2: Figure S3. Expression profiles of banana *PP2Cs*, *PYLs*, and *SnRK2s* in response to cold, salt, and osmotic treatments in BX and FJ varieties. The heat map was constructed according to the FPKM value of banana *PP2Cs*, *PYLs*, and *SnRK2s* from each replicates of two independent experiments.
